# A rare case of posterior reversible encephalopathy syndrome in a patient with severe leptospirosis complicated with rhabdomyolysis and acute kidney injury; a case report

**DOI:** 10.1186/s12879-021-06240-2

**Published:** 2021-06-03

**Authors:** Jayawardane Pathiranage Roneesha Lakmali, Kanapathipillei Thirumavalavan, Danapala Dissanayake

**Affiliations:** National Hospital of Sri Lanka, Colombo 08, Sri Lanka

**Keywords:** Leptospirosis, Posterior reversible encephalopathy syndrome, Rhabdomyolysis, Acute kidney injury

## Abstract

**Background:**

Leptospirosis is a zoonotic spirochetal disease caused by *Leptospira interrogans*. The clinical presentation ranges from an asymptomatic state to a fatal multiorgan dysfunction. Neurological manifestations including aseptic meningitis, spinal cord and peripheral nerve involvement, cranial neuropathies and cerebellar syndrome are well recognized with varying frequencies among patients with this disease. Posterior reversible encephalopathy syndrome is a very rare occurrence in leptospirosis and only two cases are reported in the medical literature up to now. We report a case of posterior reversible encephalopathy syndrome in a patient with leptospirosis with rhabdomyolysis and acute kidney injury.

**Case presentation:**

A 21 year-old male presented with fever and oliguric acute kidney injury with rhabdomyolysis. A diagnosis of leptospirosis was made and he was being managed according to the standard practice together with regular hemodialysis. The clinical condition was improving gradually. On day 8 of the illness, he developed headache and sudden painless complete bilateral vision loss followed by several brief generalized tonic clonic seizure attacks. Examination was significant for a Glasgow Coma Scale of 14/15, blood pressure of 150/90 mmHg and complete bilateral blindness. The findings of magnetic resonance imaging of the brain were compatible with posterior reversible encephalopathy syndrome. He was managed with blood pressure control and antiepileptics with supportive measures and standard treatment for leptospirosis and made a complete recovery.

**Conclusion:**

Posterior reversible encephalopathy syndrome, though very rare with leptospirosis, should be considered as a differential diagnosis in a patient with new onset visual symptoms and seizures, especially during the immune phase. Optimal supportive care together with careful blood pressure control and seizure management would yield a favourable outcome in this reversible entity.

**Supplementary Information:**

The online version contains supplementary material available at 10.1186/s12879-021-06240-2.

## Introduction

Leptospirosis is a zoonotic disease caused by the pathogenic spirochete *Leptospira interrogans*. The clinical course varies from an asymptomatic state through mild self-limiting illness to a life threatening multiorgan dysfunction and fatality. Aseptic meningitis is the commonest neurological manifestation, which is observed in 50 to 85% of cerebrospinal fluid (CSF) samples from infected individuals [1]. However, overt meningoencephalitis, transverse myelitis, myeolopathy, myeloradiculopathy, cerebellar syndrome, facial nerve palsy and Guillain-Barre syndrome, all have been reported in association with leptospirosis with varying frequencies [2]. Posterior reversible encephalopathy syndrome (PRES) is a very rare occurrence in leptospirosis. Only two case reports are published in the medical literature so far according to our knowledge [3, 4].

## Case presentation

A 21 year-old previously healthy, male university student was admitted to our ward with high fever with chills, rigors, severe myalgia for 4 days and oliguria with dark red coloured urine for 1 day. Patient gave a history of working in a paddy field about 10 days prior to the admission. On examination he was of average built with a body mass index (BMI) of 22 kg/m^2^. Temperature was 101 °F. He was tachypnoeic, not pale or icteric and no features of fluid overload was apparent. Pulse rate was 108 beats per minute and blood pressure was 110/70 mmHg. Rest of the cardiovascular and respiratory systems were normal on examination. Abdominal examination was also normal except for a severe generalized wall tenderness. His Glasgow coma scale (GCS) was 15/15 and nervous system examination including the fundi was unremarkable.

Given the typical presentation during a local outbreak period, a working diagnosis of leptospirosis complicated with possible rhabdomyolysis and acute kidney injury was made. He was started on IV Ceftriaxone 1 g twice daily according to the national guidelines after obtaining samples for relevant investigations. An urgent venous blood gas (VBG) showed severe metabolic acidosis. Serum creatinine was 528 μmol/L with blood urea (BU) of 15 mmol/L. White blood cell count (WBC) was 11.3 × 10^9^/L with 89% of neutrophils. Platelet count was 84 × 10^9^/L. He had a C-reactive protein (CRP) of 126 mg/dL. The creatine phospho kinase (CPK) level was 390,000 U/L and urine was positive for myoglobin. He underwent hemodialysis on the day of admission. Post hemodialysis serum creatinine was 260 μmol/L with BU of 11 mmol/L, and the clinical state stabilized.

He was managed according to the standard practice with antibiotics and supportive measures with a special attention to fluid balance. His vital parameters remained stable and fever gradually subsided over the next 4 days together with myalgia. However, he remained anuric and required regular hemodialysis. Serum creatinine and BU was stable in the range of 200 μmol/L and 10 mmol/L respectively and CPK level reduced steadily. *Leptospira* polymerase chain reaction (PCR) sent on admission became positive confirming the diagnosis.

**Fig. 1:**
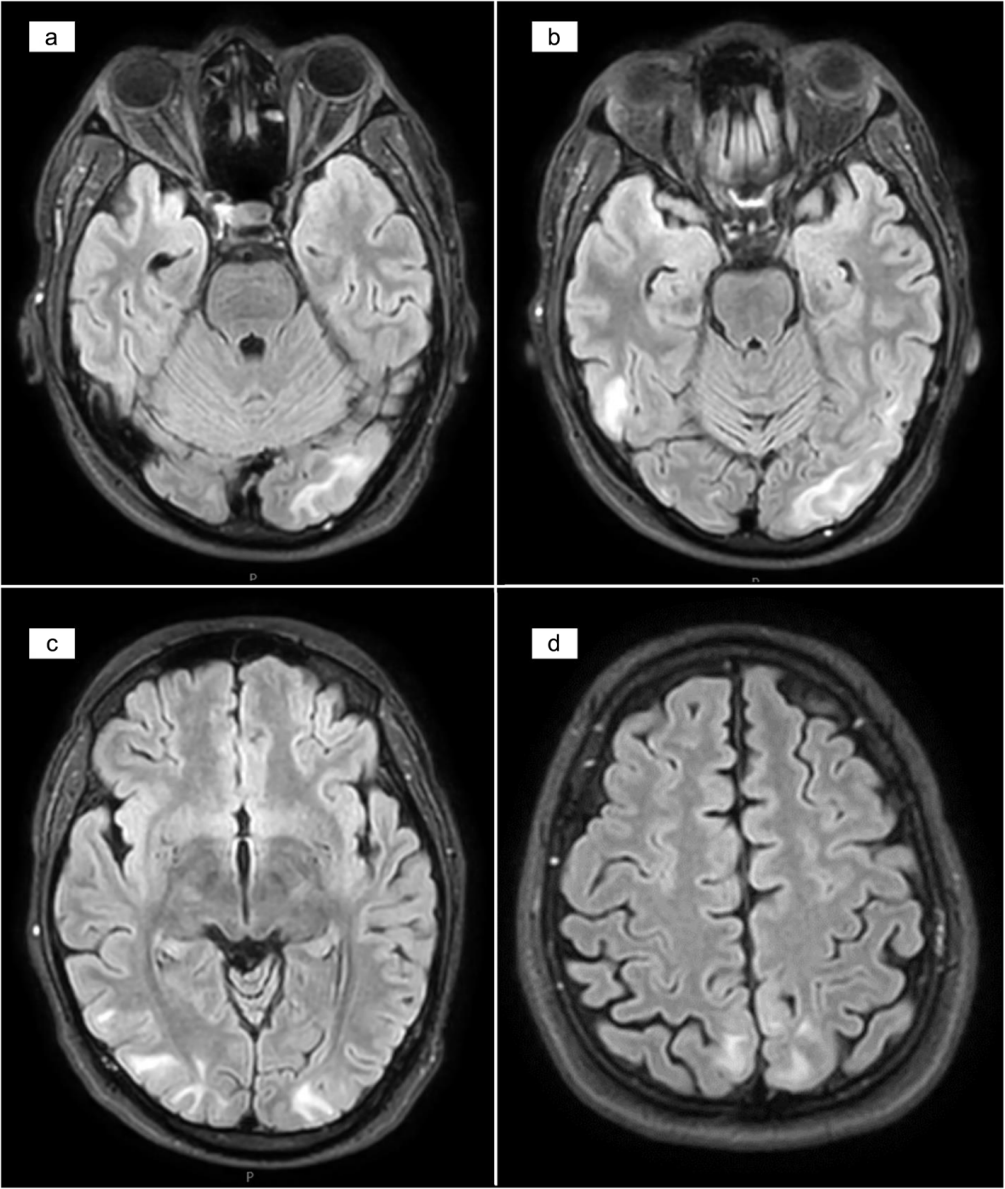
MRI T2 FLAIR images showing high signal intensities involving mainly the subcortical white matter. **a**) Left occipital area. **b**) Right temporal area. **c**) Bilateral occipito-parietal area. **d**) Bilateral parietal area

On the 4th day of inward stay, patient complained of a generalized headache and about a few hours later developed painless vision loss. There was complete blindness which was followed by several attacks of brief generalized tonic clonic seizures. He was confused and agitated in between attacks. On a limited neurological examination no abnormality was detected except for a GCS of 14/15, and complete blindness bilaterally. There was no meningism or papilloedema. Blood pressure was 150/90 mmHg and no abnormality in other system examination was apparent. Leptospirosis with encephalitis, uremic encephalopathy, acid-base or electrolyte disturbance and PRES were major differential diagnoses at this point. An urgent non contrast computed tomography (NCCT) scan of the brain done after stabilizing the patient showed no abnormality and no major acid base or electrolyte disturbances were noted in the VBG. An urgent magnetic resonance imaging (MRI) of brain with magnetic resonance arteriogram (MRA) and magnetic resonance venogram (MRV) was done which showed T2/fluid attenuated inversion recovery (T2/FLAIR) high signal intensities in the left occipito-parietal and right temporo-parietal regions, involving mainly the subcortical white matter and a small area of cortical diffusion restriction in the left occipital lobe (Fig. 1). MRA and MRV were normal. The features were consistent with PRES. [5] Lumbar puncture, though was imperative in arriving at a diagnosis of leptospirosis related encephalopathy, could not be performed due to low platelet count of 90 × 10^9^/L.

The seizures were managed with IV Levetiracetam 500 mg twice daily together with standard supportive care. IV Labetalol was administered to gradually down-titrate blood pressure, about 25% per day. With these measures together with antibiotics and hemodialysis patient recovered gradually and regained vision to his baseline level after 2 days. Repeat MRI brain could not be performed due to the limited availability of resources. Meanwhile, the microscopic agglutination test (MAT) for Leptospira done on day 10 of illness became positive for *Leptospira bakeri* with a titre of 1:320. Ceftriaxone was given for 7 days. The AKI resolved gradually, and he was taken off dialysis after 18 days of hospital stay. He was discharged after 20 days of inpatient care.

The antiepileptics were continued for 3 months as per opinion of consultant neurologist and tapered off slowly. Serum creatinine level after 3 months was 90 μmol/L. He made a complete recovery without long term renal or neurological consequences.

## Discussion

Leptospirosis is a widespread zoonosis caused by the pathogenic spirochete *Leptospira interrogans*. The disease is acquired by direct or indirect exposure to infected natural hosts, mainly rodents that carry the pathogen in proximal renal tubular epithelium. Exposure to infected urine shed by the hosts is the main mode of human infection, although human to human infection by various mechanisms has been reported rarely [6, 7].

The global burden of leptospirosis as estimated by WHO is 873,000 cases annually with 48,600 deaths [8]. Sri Lanka had an average 10,423 cases annually from 2008 to 2015 and approximately 730 annual deaths for the same period [9].

Leptospirosis is a biphasic illness with an early leptospiremic phase and a late immune phase. Toll-like receptor 2 and 4 (TLR2, TLR4), various cytokines and interleukins are implicated in the pathogenesis. High organism load during the leptospiremic phase activates the innate immune system triggering a tissue based and a systemic response that lead to organ failure and adverse outcomes. Superantigen stimulation of nonspecific T cell activation may play a role in severe forms of human leptospirosis [10].

Infection with pathogenic *Leptospira* could be asymptomatic, or give rise to a wide range of clinical presentation, varying from a mild self-limiting disease to multiorgan dysfunction and death. Renal failure with jaundice (Weil’s disease), pulmonary hemorrhage, rhabdomyolysis and myocarditis may complicate the clinical course. Aseptic meningitis is the commonest neurological manifestation, which is observed in 50 to 85% of CSF samples from infected individuals [1]. However, overt meningoencephalitis, transverse myelitis, myelopathy, myeloradiculopathy, cerebellar syndrome, facial nerve palsy and Guillain-Barre syndrome, all have been reported in association with leptospirosis with varying frequencies [2]. PRES is a very rare occurrence in leptospirosis and only two cases are reported up-to-date. Aram J from United Kingdom published the first case in medical literature in 2010, of a male who developed PRES during the recovery phase of the illness after 3 weeks of antibiotic therapy [4]. Priyankara WD, from Sri Lanka published the second case in 2019, of a 29 year old male with severe leptospirosis with myocarditis, pulmonary hemorrhages and acute kidney injury in whom the PRES occurred during the early immunologic phase [3]. The two patients described in above reports and the patient we describe here demonstrate certain similarities. All are young males and all of them are without prior medical co-morbidities. All three patients have developed severe leptospirosis, especially with renal dysfunction. The infecting serovar in our patient and the other patient from Sri Lanka is *Leptospira bakeri*. The diagnosis of PRES was made in the immunologic phase in all three cases and all of them made uneventful recovery.

PRES is a clinical syndrome of heterogenous etiologies characterized by headache, altered level of consciousness, visual defects and seizures. The pathogenesis appears to be related to endothelial dysfunction and disorders of cerebral autoregulation resulting in leakage of fluid and vasogenic edema. PRES characteristically involves the subcortical white matter of posterior cerebral hemispheres, although other regions of the brain may also be affected. The predilection to the posterior circulation is partially explained by the lack of sympathetic innervation of the vertebro-basilar system when compared to the anterior circulation [11]. A wide variety of conditions are implicated as etiologies in PRES including hypertensive encephalopathy, acute and chronic renal diseases and vasculitides [12].

The pathogenesis of PRES in leptospirosis has not been widely studied. However, secondary immune mediated capillary endothelial damage with transient hypertension is proposed as a possible mechanism by previously reported two case reports where both patients developed PRES during the immune phase.

Leptospirosis in its severe forms is thought to be a form of systemic vasculitis. This was demonstrated in autopsy studies where aortitis and coronary arteritis were described. Toxins and various antigens released during the lysis of *Leptospira* with treatment are also postulated as culprits of endothelial dysfunction. However, the exact mechanism of vascular injury is as yet unexplained [13].

Management of PRES is symptomatic since there are no specific treatment modalities. Management of hypertension and seizures, together with the specific therapy for the underlying condition is the routine practice. There are no controlled trials to determine the best approach in this regard [14].

Our patient also developed symptoms after about 2 weeks of initial infection, during the early immune phase. He had acute kidney injury with transient mild hypertension at the time of symptom onset which we believe to have precipitated the occurrence of PRES. Severe rhabdomyolysis as seen in our patient has been associated with PRES in published literature [15, 16]. Although, our patient had a decremental trend of CPK at the time of onset of symptoms with an absolute value of 80,000 U/L, we believe the still high CPK value might have been another contributory factor in this unique case.

## Conclusion

Posterior reversible encephalopathy syndrome is a very rare neurological manifestation of severe leptospirosis. It should be considered as a differential diagnosis in a patient with leptospirosis with visual symptoms and seizures, especially during the immune phase. Optimal supportive care together with careful blood pressure control and seizure management would yield a favourable outcome in this reversible entity.

## Supplementary Information

**Additional file 1.** Timeline is attached as supplementary material.

## Data Availability

All data generated or analysed during this study are included in this published article.

## Abbreviations

BMI: Body mass index
BU: Blood urea
CPK: Creatine phospho kinase
CRP: C-reactive protein
CSF: Cerebro spinal fluid
FLAIR: Fluid attenuated inversion recovery
GCS: Glasgow Coma Scale
MAT: Microscopic agglutination test
MRA: Magnetic resonance arteriogram
MRI: Magnetic resonance imaging
MRV: Magnetic resonance venogram
NCCT: Non contrast computed tomography
PCR: Polymerase chain reaction
PRES: Posterior reversible encephalopathy syndrome
TLR 2: Toll-like receptor 2
TLR 4: Toll-like receptor 4
VBG: Venous blood gas
WBC: White blood cell count
WHO: World Health Organization
