# Supplementary figures and images for: A rare case of posterior reversible encephalopathy syndrome in a patient with severe leptospirosis complicated with rhabdomyolysis and acute kidney injury; a case report
Source: BMC Infect Dis. 2021 Jun 3;21:522. doi: 10.1186/s12879-021-06240-2 (PMC8176595; doi:10.1186/s12879-021-06240-2)

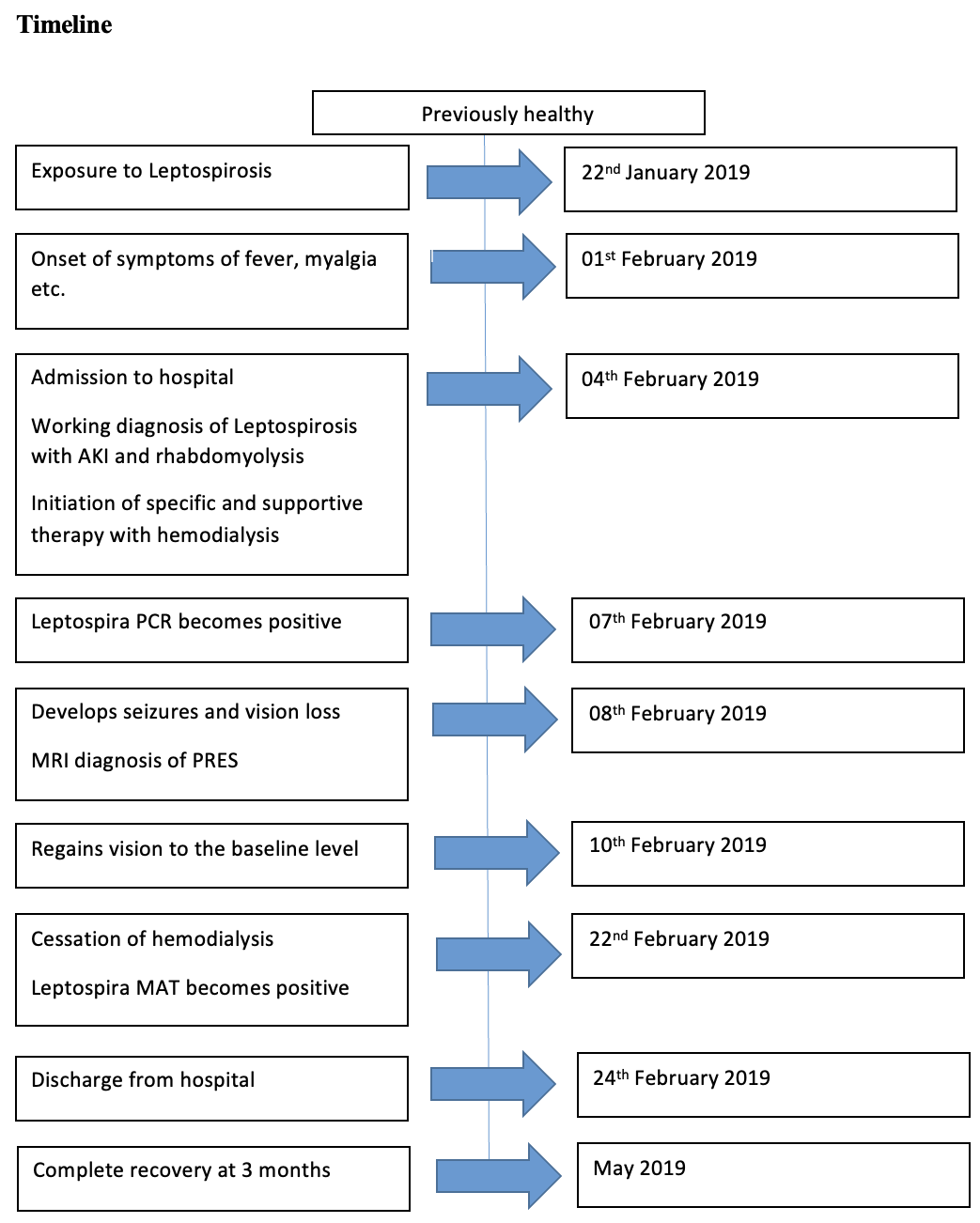

Supplement: Supplementary file 1 — Additional file 1. Timeline is attached as supplementary material. [file 12879_2021_6240_MOESM1_ESM.docx]
